# The regulation of protein translation and its implications for cancer

**DOI:** 10.1038/s41392-020-00444-9

**Published:** 2021-02-18

**Authors:** Ping Song, Fan Yang, Hongchuan Jin, Xian Wang

**Affiliations:** 1 Department of Medical Oncology, Cancer Institute of Zhejiang University, Sir Run Run Shaw Hospital, Zhejiang University School of Medicine, Hangzhou, Zhejiang China; 2 Department of Hepatobiliary and Pancreatic Surgery, First Affiliated Hospital, Zhejiang University School of Medicine, Hangzhou, Zhejiang China; 3 Key Lab of Biotherapy in Zhejiang, Sir Run Run Shaw Hospital, Zhejiang University School of Medicine, Hangzhou, Zhejiang China

**Keywords:** Non-coding RNAs, Oncogenes

## Abstract

In addition to the deregulation of gene transcriptions and post-translational protein modifications, the aberrant translation from mRNAs to proteins plays an important role in the pathogenesis of various cancers. Targeting mRNA translation are expected to become potential approaches for anticancer treatments. Protein translation is affected by many factors including translation initiation factors and RNA-binding proteins. Recently, modifications of mRNAs mainly N6-methyladenine (m^6^A) modification and noncoding RNAs, such as microRNAs and long noncoding RNAs are involved. In this review, we generally summarized the recent advances on the regulation of protein translation by the interplay between mRNA modifications and ncRNAs. By doing so, we hope this review could offer some hints for the development of novel approaches in precision therapy of human cancers.

## Introduction

**Fig. 1:**
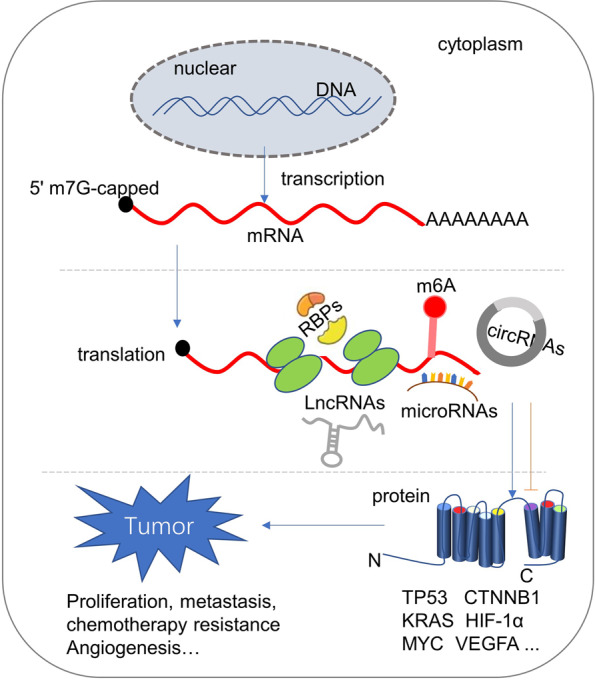
The process of gene expression. Gene expression needs to be transcribed from DNA to RNA, translated into protein, and modified into mature protein to perform biological functions. This figure highlights the influence of RBPs, microRNAs, lncRNAs, circRNAs, and RNA m6A modification on the protein translation procedure in tumorigenesis

Cancer is a major global public health problem and the second leading cause of death worldwide.^1^ It has been recognized as a disease resulting from the accumulation for multiple genetic and epigenetic changes. Genetic mutations mainly alter the functions of corresponding proteins, while epigenetic changes will change the expression of potential oncogenes and tumor suppressor genes. The regulation and function of these alterations have been substantially explored.^2–7^ However, the relevance of protein translation or the production of nascent proteins from mRNAs to the initiation and progression of human cancers used to be largely overlooked. With the recent development of experimental technologies, the regulation of protein translation has been gradually revealed. It could be affected by various factors including well-recognized players such as translation initiation factors and RNA-binding proteins, as well as new players such as noncoding RNAs and m6A modification of mRNAs (Fig. 1). In this review, we aimed to summarize the new advances in the regulation of protein translation, especially the contribution of dysregulated ncRNAs to the aberrant protein translation in cancers and facilitate a better understanding of the mechanism of carcinogenesis.

## Overview of the protein translation process

### Translation steps

In general, protein translation can be divided into three main steps: initiation, extension, and termination. Initiation is the rate-limiting step in translation, and the formation of the translation initiation complex (eIF4F) is the most critical process.^8^ eIF4F consists of eIF4A, eIF4E, and eIF4G. Among them, eIF4A is a helicase that expands RNA into a single strand through the RNA-binding protein eIF4B. eIF4E is a cap-binding protein and eIF4G is a scaffolding protein used for mechanical assembly. eIF4F assembles on the structure of 5′ m7G-capped of RNA and interacts with polyA tail-binding protein (PABP). However, the cap-independent mRNA translation process, which is IRES (internal ribosome entry site)-dependent translation, is triggered when the mTOR signaling pathway is inhibited by rapamycin, nutritional deficiencies, or hypoxia. As a result, activated 4EBP1 (dephosphorylation) binds to eIF4E and inhibits cap-dependent translation. Meanwhile, eIF4G expression increases and mediates IRES-dependent translation. After the translation initiation complex is assembled, the 40S ribosomal subunit binds to other initiation factors, including eIF5 and the ternary complex (eIF2-GTP-Met-tRNAi) to form a 43S ribosome, thus enabling the progression into the extension stage.^9,10^ The initiation complex of cap-dependent translation scans the mRNA from the 5 ‘end to find the first start codon (AUG), and enters the ribosomal P-site via initiator methionyl tRNA (Met-tRNAi). After that, the 43S ribosome was recruited to the 5′ cap structure of mRNA to form a 48S ribosomal complex via eIF3 complex interacting with eIF4G. Meanwhile, GTP bound by eIF2 (composed of α-, β-, and γ subunits) is activated by hydrolysis, leading to the release of eIF2-GDP after 60S ribosomes are recruited, leaving the active 80S ribosomes (40S and 60S subunits) to enter the extension phase.^11,12^

**Fig. 2:**
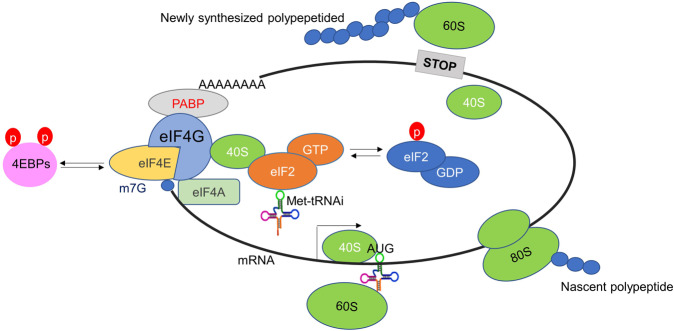
The model of protein translation process. Translation is initiated by eIF4F assembly, consists of eIF4A, eIF4E, and eIF4G. The hypophosphorylated form of 4E-BPs competes with eIF4G to bind eIF4E, and phosphorylated eIF2α fails to recover the GTP required for Met-tRNAi, resulting in failure of the formation of eIF4F complexes and inhibits translation. Extension: the initiation complex of cap-dependent translation scans the mRNA from the 5 ‘end to find the first start codon (AUG), and enters the ribosomal P-site via initiator methionyl tRNA (Met-tRNAi). Termination: when the mRNA with stop codon (UAG, UGA, UAA) enters the ribosomal A-site, it can’t be recognized by activated amino acid (aa-tRNA), the translation process is finished

Termination: when the mRNA with stop codon (UAG, UGA, UAA) enters the ribosomal A-site, it can’t be recognized by activated amino acid (aa-tRNA). Instead, the protein releasing factor binds to the A-site, leading to the termination of protein synthesis^13^ (Fig. 2).

### Translation components

In addition to mRNA as a template for protein translation, ribosomal rRNA is required to provide translation sites, and transfer RNA (tRNA) as a tool for amino acid transport.^14,15^ tRNA carries specific amino acids to the ribosome under the action of aminoacyl tRNA synthetase, and accurately recognizes the codon sequence on mRNA by its anticodon, and adds specific amino acids into the peptide.^16^ Aberrant expression of rRNA and tRNA during tumorigenesis is frequently found in different tumors, including colorectal cancer,^17^ rhabdomyosarcoma,^18^ and retinoblastoma.^19^ There are only 61 types of tRNAs involved in the process of protein translation in eukaryotic cells. Due to the complexity of the secondary structures of tRNAs, it is difficult to design the primer and the research on tRNAs has been greatly restricted. However, with the development of high-efficiency reverse transcriptase enzymes,^20^ the exploration of tRNAs such as tRNA sequencing and quantification gradually became possible and popular.

### The role of translation initiation factors in carcinogenesis

Increased abundance of components of translation initiation complex is prevalent in various kinds of tumors.^21^ For example, in BRAF-mutated tumors, increased expression of eIF4F complex leads

to anti-BRAF therapy resistance and more metastasis. Blocking the eIF4E-eIF4G interaction or targeted-inhibition of eIF4A synergized BRAF-targeting therapy. Therefore, eIF4F can be used as an indicator of acquired resistance to BRAF (V600) targeting therapy.^22^

The binding of eIF4E to the 5′ m7G-capped of the mRNA is the rate-limiting step for translation initiation. The genome-wide translation profiling reveals that the content of eIF4E is crtical for the translation of mRNAs that regulate the reactive oxygen species (ROS), which promotes cancer cell survival.^23^ By degrading the mRNA of eIF4E-BP2, which negatively regulates eIF4E, IGF2BP3 promotes eIF4E-mediated translation activation, and ultimately leading to tumor cell proliferation.^24^ Phosphorylated eIF4E promotes protein translation and also plays an important role in antitumor immunotherapy. In a liver cancer model induced by MYC overexpression combined with KRAS mutation (G12D), cancer cells escape immune surveillance and suppress the cytotoxicity of CD8 + T cells by increasing PD-L1 protein translation. While, eFT508, in phase 2 clinical trials (NCT02937675, NCT02605083 and NCT03258398), inhibits eIF4E phosphorylation to reduce the translation of PD-L1 protein, reverses the aggressive and metastatic characteristics.^25^ In summary, activated eIF4E promotes the RNA translation to accelerate the development of cancer by enhancing proliferation, inducing resistance to chemotherapy and anti-immune therapy. Encouragingly, there are small-molecule inhibitors available to target eIF4E in the clinical trials, such as eIF4E antisense oligonucleotides (ASOs), in phase 2 clinical trials (NCT02605083), targeted the eIF4E mRNA for destruction, proven to inhibit tumor growth.^26,27^

The hypophosphorylated form of eIF4E-binding proteins (4E-BPs) competes with eIF4G to bind eIF4E, which affects the formation of eIF4F complex and inhibits translation. 4E-BPs inhibit the translation of proliferation and cell cycle proteins to affect tumor cell proliferation and growth.^28,29^ As a small-molecule inhibitor of the eIF4E/eIF4G interaction, 4EGI-1 can mimic 4E-BP function to restrain cap-dependent translation and inhibit the growth of multiple cancer cell lines,^30^ highlighting the potential for targeting protein translation in the design of novel approaches in cancer treatment.

eIF2α plays an important role in the process of translation initiation. Once phosphorylated, it loses its activity to recover the GTP required for Met-tRNAi, resulting in translation inhibition. Smad7 promotes the proliferation of colon cancer cells by activating eIF2α to increase CDC25 protein translation, a phosphatase that dephosphorylates CDK2, controls progression of the cell cycle through the S phase.^31^ In melanoma, the tumor suppressor gene PTEN increases the phosphorylation of eIF2α via PDZ binding motif, promoting tumor cell apoptosis and inhibiting tumor proliferation.^32^ In prostate cancer, proteasome inhibitors (PIs) reduce the level of HIF1a protein translation by inducing eIF2α phosphorylation, to inhibit the transcriptional activity of HIF1a, and downregulate the expression of target gene VEGF, thereby inhibiting angiogenesis.^33^ However, whether phosphorylated eIF2α serving as protumor or antitumor role remains controversial, depending on the particular proteins affected on different tumor types and grades. There are studies indicating that increased eIF2α phosphorylation may promote cancer development by enhancing the translation of target mRNAs. For example, decreased expression of PKR (also known as EIF2AK2), which is an eIF2α phosphatase, is associated with less aggressive human cancers.^8^ GCN2 phosphorylates eIF2α, promotes ATF4 protein translation by delayed restart (two uORFs in mRNA leader),^34^ which enhances tumorigenicity of fibrosarcoma cells in nude mice.^35^ Because of the paradoxical functions of eIF2α, directly inhibiting phosphorylation need to consider the specific microenvironment of tumors.

## The effects of post-transcriptional regulation on protein translation in tumors

### RNA-binding protein

As the most widely studied post-translational regulators, RNA-binding proteins (RBPs) are involved in all aspects of RNA biological processes, including RNA metabolism, transport, and localization. Mechanistically, it has been speculated that RBPs reshape the structure of certain mRNA to change the affinity for translation machinery, thus affecting its translation.^36^ However, the exact mechanism still needs to be explored and confirmed. Functionally, they play a pivotal role in regulating the RNA translation in tumorigenesis^37^ (Table 1). For example, Musashi-1 (MSI1) and Musashi-2 (MSI2) are highly expressed in a variety of tumors, and they can functionally inhibit or promote the translation of both essential oncogenes and tumor suppressor genes through their N-terminal RNA recognition sequence (RRM).^38^ Kawahara et al. found that MSI1 competed with eIF4G to bind PABP to inhibit translation of downstream genes to maintain the stem cell status.^39^ Sun-Mi et al. found MSI2 controlled efficient translation of Hoxa9, Myc, and Ikzf2 mRNAs to maintain the mixed-lineage leukemia (MLL) self-renewal program.^40^ In a research carried by Li and his colleagues, eIF3 was highly expressed in liver cancer, playing an important role in the initiation of translation and promotes the development of cancer.^41^ In leukemia, SYNCRIP is a new RBP that promotes HOXA9 translation and controls myeloid leukemia stem cell programs.^42^ CELF1 (CUGBP Elav-like family member 1), inhibits MYC protein translation to reduce the renew and proliferation of small intestinal epithelial cells.^43^ In the colorectal cancer, HuR (ELAV-like RNA-binding protein 1), binds to the 5′UTR region of the proapoptotic gene caspase-2L, inhibiting its translation, and promoting tumor cell antiapoptosis activity.^44^ In RKO intestinal cancer cells of wild-type p53, under short-term UV irradiation, HuR can promote P53 translation.^45^ Moreover, HuR inhibits wnt-5a protein translation in breast tumors.^46^ Meanwhile, there are reports indicating that CPEBs (cytoplasmic polyadenylation element binding protein) induce cytoplasmic polyadenylation with specific polyA polymerase to enhancing translation and facilitating tumorigenesis and metastasis.^47^ IMP-3, a member of the insulin-like growth factor II (IGF-II) mRNA-binding protein (IMP) family, promotes IGF-II protein translation to induce the tumor cell proliferation in leukemia.^48^ When IFN-γ is used to treat inflammation in bone marrow cells, GAIT complex inhibits VEGFA translation by binding to the 3′UTR of VEGFA, while under hypoxic conditions, hnRNP L (heteroribonucleoprotein) is activated, interacting with DRBP76 (interleukin enhancer binding factor 3) and hnRNP A2/B1 to form a heterotrimer, which blocks GAIT-mediated translation inhibition, and promotes VEGFA expression.^49^ Based on these studies, it is evident that RBPs participate in tumor stemness, metastasis, proliferation, and immunity by influencing the translation of target genes. However, as RBPs are nonenzymatic, indicating the lack of hydrophobic pockets capable of binding small-molecule inhibitors, they are difficult to be therapeutic targets.

**Table 1:** RNA-binding proteins involved in translation regulation

RBPs	Protein	Clinical correlates in cancers	Ref
MSI1	Inhibits downstream genes	Maintains the stem cell status	^40^
MSI2	Promotes Hoxa9, Myc, and Ikzf2	Maintains the self-renewal program of MLL	^41^
SYNCRIP	Promotes HOXA9 translation	Control myeloid leukemia stem cell program	^43^
CELF1	Inhibits MYC translation	Inhibits the renew and proliferation of small intestinal epithelial cells	^44^
HuR	Inhibits Caspase-2L translation	Promotes antiapoptosis of the colorectal cancer cell	^45^
HuR	Promotes P53 translation	In intestinal cancer cells	^46^
CPEBs	Inhibits wnt-5a translation regulates polyadenylation	In breast cancer	^47^
IMP-3	and resulting translation	Facilitates tumorigenesis and metastasis	^48^
GAIT	promotes IGF-II translation	Promotes tumor proliferation in leukemia	^49^
	inhibits VEGFA translation	Bone marrow cells under immunotherapy	^50^

### Noncoding RNAs

The noncoding RNA described in this review mainly includes small RNA (microRNA), long noncoding RNA (lncRNA), and circular RNA (circRNA), which have an important effect on tumors.^50^ In Table 2, we summarized the effects of noncoding RNA on translation procedure in tumors.

**Table 2:** Noncoding RNAs involved in translation regulation

	Protein	Clinical correlates in cancers	Ref.
**microRNA**			
miR-10b	inhibits HOXD10 translation	promotes the migration and invasion of breast cancer	^54^
miR-12528	inhibits IGF1R translation	inhibits cell proliferation and metastasis in lung cancer	^55^
miR-146a	inhibits c-met translation	inhibits colorectal cancer liver metastasis	^56^
miR-125b	inhibits PIGF translation	inhibits the angiogenesis of liver cancer	^57^
miR-143-3p	inhibits BRD2 translation	increases the therapy sensitivity to cisplatin in gastric cancer	^58^
miR-648	inhibits MGMT translation	sensitizes gliomas to temozolomide treatment	^59^
miR-675-5p	inhibits TP53 translation	promotes the development of colorectal cancer	^60^
miR-10a	promotes RP translation	promotes the overall protein translation	^61^

**microRNA&RBP**			
miR-143 and MSI2	regulates KRAS protein translation	affects the tumor growth of bladder cancer	^62^
miR-155-5p and HuR	promotes HuR translation	supports the metastasis of colorectal cancer	^63^
miR-330 and HuR	regulates STAT3 translation	cachexia	^64^
miR-17-19b and HuR	regulates the translation of MYC	ensure the optimal growth of B-cell lymphoma	^65^

**LncRNA**			
lncRNA-GMAN	promotes ephrin A1 translation	enhances the invasion ability of gastric cancer	^70^
lncRNA-GMAN	regulates BCL-2, BCL-6, XIAP, e-cadherin, n-cadherin, vimentin, and snail translation	promotes the development of liver cancer	^71^
lncRNA-MALAT1	increases TCF7L2 translation	promotes aerobic glycolysis in liver cancer	^72^
LncNB1	promotes E2F1 translation	induces neuroblastoma cell proliferation and survival.	^73^
RP1-5O6.5 (RP1)	attenuates p27kip1 translation	promotes breast cancer cell proliferation and metastasis	^74^
PYCARD-AS1	regulates PYCARD translation	antiapoptosis of breast cancer	^75^

	Protein	Cancer	Ref.
**LncRNA and RBP**			
OCC-1 and HuR	regulates the translation of HuR targets	colorectal cancer	^76^
7SL and HuR	attenuates P53 translation	HeLa	^77^
lincRNA-p21 and HuR	regulates the translation of JUNB and CTNNB1	cervical carcinoma	^78^
lncRNA-TRMP and PTBP1	regulates P27 translation	A549 cell	^79,80^
AFAP1-AS1 and AUF1	promote translation of ERBB2	breast cancer	^81^
Lnc-LBCS and hnRNPK	regulates AR translation	prostate cancer	^82^
lncRNA-HITT and YB-1	regulates HIF-1α translation	colon cancer	^83^

**LncRNA and microRNA**			
LINC00460 and miR-149-5p	regulates CUL4A translation	colorectal cancer	^84^
LINC00346 and miR-34a-5p	regulates the translation of CD44, NOTCH1, and AXL proteins	gastric cancer	^85^
HOXA11-AS and miR-1297	regulates EZH2 protein translation	gastric cancer	^86^
lncRNA H19 and miR-29b-3p	regulates MCL-1 translation	myeloma	^87^

**circRNA**			
circβ-catenin	generates a new 370 amino acid β-catenin isoform	liver cancer	^90^
Circ-FBXW7	encodes a new 21-kDa protein called FBXW7-185aa	gliomas	^91^
circ-SHPRH	translates a new 17 kDa protein, SHPRH-146aa	gliomas	^92^
circYap	inhibit the translation of Yap protein	breast cancer	^93^
circPABPN1 and HuR	regulates the translation of PABPN1	Hela	^95^
OIP5-AS1 and HuR	regulates target genes of HuR	—	^96^
circRNA-MTO1 and TRAF4	inhibits the translation of Eg5	breast cancer	^97^
circ-MALAT1 and miR-6887-3p	enhances JAK2 expression	liver cancer	^98^
circEIF4G2 and miR-218	increases HOXA1 translation	cervical cancer	^99^

### microRNA

MicroRNA is small noncoding RNA containing about 22 nucleotides. By complementary base pairing, microRNAs bind to the 3′UTR region or 5′UTR region of mRNA, thus mediating the degradation or translation of target mRNAs.^9^ It’s widely recognized that miRNAs inhibit cap-dependent translation at the initiation step by dissociating the translation initiation factors from targeted mRNAs.^51,52^ Over the past decades, a bunch of researches demonstrated that microRNA plays an important role in tumorigenesis and development. In terms of influencing the translation process alone, microRNA-10b (miR-10b) is highly expressed in metastatic breast cancer. miR-10b inhibits HOXD10 translation via interaction with its 3′UTR and promotes the migration and invasion.^53^ miR-12528 inhibits insulin-like growth factor 1 receptor (IGF1R) translation to inhibit cell proliferation and metastasis in lung cancer.^54^ miR-146a inhibits tumor formation, and colorectal cancer liver metastasis by limiting c-Met translation.^55^ The expression of miR-125b is low in liver cancer tissue comparing to normal liver tissue, and overexpression miR-125b can inhibit the translation of placental growth factor (PIGF) protein to reduce the angiogenesis.^56^ Comparing to normal gastric tissue, the expression of mir-143-3p is significantly lower in gastric cancer tissues. Overexpression of mir-143-3p inhibits BRD2 protein translation, thereby inhibiting cancer cell proliferation and increasing the therapy sensitivity to cisplatin.^57^ In glioma, miR-648 inhibits O6-methylguanine-DNA methyltransferase (MGMT) protein translation, reducing the production of MGMT and thus sensitizing MGMT-expressing gliomas to temozolomide treatment.^58^ In colorectal cancer, overexpressed prostaglandin E2 (PGE2) upregulates miR-675-5p expression to inhibits TP53 translation, thus promoting tumor development.^59^ In a research carried out by Orom et al., miR-10a could bind to the 5′UTR of ribosomal protein (RP) mRNA to promote the translation under amino acid starvation conditions, and regulated the overall protein translation.^60^ In conclusion, microRNAs can inhibit the proliferation, metastasis, angiogenesis of tumors and sensitize tumors to chemotherapy drugs by decreasing the protein synthesis of certain target genes.

MicroRNAs can also interact with RBPs to jointly regulate protein translation. For example, miR-143 inhibits MSI2 protein translation through 3′UTR base pairing to reduce the translation of KRAS which interacts with MSI2, thereby inhibiting tumor growth in bladder cancer.^61^ In colorectal cancer, MiR-155-5p promotes HuR translation to support cell metastasis.^62^ In tumor-induced cachexia, HuR promotes translation of STAT3 mRNA by preventing miR-330-mediated translation inhibition.^63^ In B-cell lymphoma, miR-17-19b indirectly reduces the translation efficiency of MYC by downregulating Chek2 and increasing the binding of HuR to MYC mRNA.^64^

Taken together, these studies highlight the balance between RBPs and microRNAs has an important impact on the translation of target genes. Indeed, antitumor therapeutics targeting microRNAs by miRNA mimics and inhibitors have shown promise in preclinical development.

### LncRNA

LncRNAs contain about 200 nucleotides,^65^ and play an important role in tumor progression by regulating the chromatin organization, transcription, mRNA stability, protein translation, and post-translational modification.^66–68^ As the scaffold of nuclear bodies involved in the translation regulation, lncRNAs play a crucial part in gene expression.

Gastric cancer metastasis-associated long noncoding RNA (GMAN) promotes the translation of ephrin A1 by binding with antisense GMAN RNA (GMAN-AS), enhancing the invasion ability of gastric cancer cells.^69^ Furthermore, it is highly expressed in liver cancer and interacts with eIF4B. By inhibiting the dephosphorylation of PPP2R2A, it promotes phosphorylation of serine 422 at eIF4B, and subsequent phosphorylated eIF4B increases the antiapoptotic proteins BCL-2, BCL-6, XIAP and migration-related proteins e-cadherin, n-cadherin, vimentin, and snail translation to promote the development of liver cancer.^70^ lncRNA-MALAT1 increases TCF7L2 translation by activating the mTOR-4EBP1 signal axis and SRSF1, and promotes aerobic glycolysis in liver cancer.^71^ LncNB1 is overexpressed in MYCN-amplified neuroblastoma and induces neuroblastoma cell proliferation and survival. In mechanism, lncNB1 promotes the transcription of DEPDC1B and translation of E2F1 mRNA, leading to phosphorylation of N-MYC and stable protein expression.^72^ RP1-5O6.5 (RP1) is highly expressed in breast cancer and promotes the proliferation and metastasis of breast cancer cell. The combination of RP1 and p-4E-BP1/eIF4E can prevent eIF4E from interacting with eIF4G, thus attenuating the translation of p27kip1 mRNA, and increasing the expression of snail to improve the invasiveness.^73^ In breast cancer cells, PYCARD-AS1, the antisense lncRNA of proapoptotic gene PYCARD, overlaps and interacts with PYCARD at the 5 ′ end, inhibiting ribosome assembly in the cytoplasm to limit the translation efficiency of PYCARD.^74^

LncRNAs also play a role in protein translation by interacting with RBPs. LncRNA OCC-1 plays as suppressor in colorectal cancer. By binding to the E3 ligase β-TRCP, it increases the ubiquitination of HuR, which leads to a decrease in the expression of downstream genes.^75^ LncRNA 7SL, highly expressed in tumor cells, attenuates the translation of the tumor suppressor protein P53 by binding to the 3′UTR of P53 mRNA and squeezing HuR binding.^76^ On the other hand, HuR recruits let-7/Ago2 to lncRNA-p21, reducing its stability. The decreased binding of lncRNA-p21 to mRNAs of JUNB and CTNNB1 promotes their protein translation in HeLa cells.^77^ LncRNA-TRMP promotes cancer cell proliferation and G1/S cycle progression when P53 is activated, the authors test the cycle-related proteins and find that TRMP negatively regulates P27. By inhibiting PTBP1, P27 mRNA-binding protein, TRMP ultimately inhibits IRES-dependent translation of P27 protein.^78,79^ In terms of chemotherapy response, the expression level of actin filament-related protein 1 antisense RNA 1 (AFAP1-AS1) is higher in trastuzumab-resistant cells than that in sensitive cells. Exosomal AFAP1-AS1 can induce trastuzumab resistance. Mechanically, AFAP1-AS1 interacts with the AUF1 to promote translation of ERBB2, which is used to represent the RNA encoding the HER-2 protein.^80^ Lnc-LBCS expression is low in castration-resistant prostate cancer (CRPC) cell lines and tissues. In the absence of androgens, knockdown of LBCS is sufficient to activate androgen receptor (AR) signaling, consequently increasing AR protein translation. In terms of mechanism, LBCS directly interacts with hnRNPK to inhibit the form of hnRNPK-AR complex and thus reduces AR translation.^81^ The low expression of HITT (translation level HIF-1α inhibitor) is related to the advanced stage of colon cancer. After the expression is restored, HITT inhibits tumor angiogenesis and tumor growth. Mechanistically, the direct binding of HITT and YB-1 deceives and prevents YB-1 from binding to 5′UTR of HIF-1α mRNA, which relieves the translation promotion effect of YB-1on HIF-1α.^82^

In addition, LncRNAs can also serve as competitive endogenous RNAs (ceRNAs) to sponge or decoy on microRNAs to regulate protein translation. For example, LINC00346 recruits argonaute 2 (Ago2), which acts as a molecular sponge, antagonizes miR-34a-5p to inhibit the translation of CD44, NOTCH1, and AXL proteins, and promotes the development of gastric cancer.^83^ LINC00460 is highly expressed in colorectal cancer cells. As ceRNA, it antagonizes miR-149-5p to inhibit cullin 4A (CUL4A) translation and affects cell growth and apoptosis.^84^ HOXA11-AS acts as a sponge of miR-1297, antagonizing its ability to inhibit the translation of EZH2 protein and increasing EZH2 protein into the nucleus to inhibit the transcription of PRSS8 and KLF2. In conclusion, HOXA11-AS/miR-1297/EZH2 crosstalk promotes the proliferation, migration and invasion of gastric cancer cell.^85^ LncRNA H19 is highly expressed in multiple myeloma and promotes tumor cell growth and bortezomib resistance via inhibiting miR-29b-3p expression and enhancing MCL-1 translation.^86^

### circRNA

With the development of high-throughput transcriptome sequencing and computational methods, a bunch of researches reveal that a large amount of circular RNAs (circRNAs) are endogenous, conservative, and stable in eukaryotic cells.^87^ CircRNAs are a new type of noncoding RNA, characterized with covalent closed loop formed by reverse splicing of precursor mRNA. Recent studies have demonstrated the relation between circRNAs and polysomes. Some circRNAs contain the start codon AUG and the recognized open reading frame (ORF), indicating that circRNAs have a potential protein coding function.^88^ Circβ-catenin, abundantly expressed in liver cancer, generates a 370 amino acid β-catenin isoform, which utilizes the start codon as linear β-catenin RNA transcription and translation ends with a new termination caused by circularization codon. This newly born β-catenin can resist GSK3 phosphorylation, and finally stabilize expression and activate WNT signaling pathway.^89^ Circ-FBXW7 is highly expressed in the normal human brain. It relies on the IRES to cross the open reading frame and encode a 21 kDa protein, FBXW7-185aa. In gliomas, the upregulation of FBXW7-185aa inhibits proliferation and cell cycle.^90^ In addition, circ-SHPRH can also translate a 17 kDa protein, SHPRH-146aa, through IRES-driven open reading frames (ORF), and both are low expressed in gliomas. Overexpression of them can inhibit gliomas development.^91^

In addition to being able to regulate translation by itself, circRNAs can also regulate other protein translation by regulating translation initiation. For example, overexpression of ciri-Yap inhibits the assembly of the translation initiation complex (eIF4G and PABP), thus inhibiting the translation of Yap protein. As a consequence, overexpression of circYap inhibits the proliferation, migration, and colony formation of breast cancer cells.^92^. Furthermore, circRNAs interact with RBPs or act as ceRNA through sponge microRNAs to affect translation and play an important role in tumorigenesis and development.^93^ In HeLa cells, circPABPN1 (hsa_circ_0031288) prevents HuR from binding to PABPN1 mRNA and inhibits the translation of PABPN1 protein.^94^ Similarly, OIP5-AS1 inhibits tumor cell proliferation by preventing HuR from binding to target genes.^95^ In breast cancer, circRNA‑MTO1 (hsa‑circRNA-007874) binds to the tumor necrosis factor receptor-associated factor 4 (TRAF4), thereby inhibiting the translation of mitogenic kinesin (Eg5) and reversing Monastrol (kinesin inhibitor) resistance.^96^ circ-MALAT1 combines with ribosomes and tumor suppressor gene PAX5 to form a complex, hindering the translation of PAX5 and helping maintain cancer stem cells characteristics. It is also found that circ-MALAT1 promotes liver cancer stem cell self-renewal by acting as the miR-6887-3p sponge, enhancing JAK2 expression and activating the JAK2/STAT3 signaling pathway.^97^ In cervical cancer, highly expressed circEIF4G2 acts as a sponge for miR-218 which promotes HOXA1 protein translation, and consequently promotes cancer cell proliferation and migration.^98^

### 6-methyladenine (m6A) modification

**Fig. 3:**
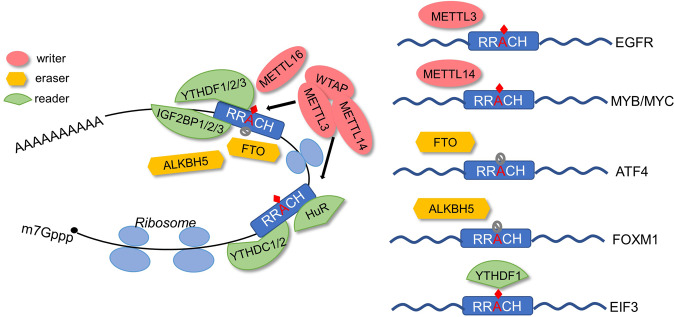
m^6^A modification regulates protein translation. The m^6^A modification is catalyzed by the writers (METTL3/METTL14/WTAP, WETTL16) and demethylated by erasers (FTO, ALKBH5). The m^6^A modification is recognized by the readers (YTHDF1/2/3, YTHDC1/2, IGF2BP1/2/3, and HuR). The translation procedure of target genes can be influenced by the m6A modification level

The m6A modification of RNA, as the most common mRNA modification in mammalian cells, usually occurs at the consensus sequence 5′-RRACH-3 ‘(R = A or G; H = A/C/U).^99^ A bunch of recent studies about m6A modification have been performed in tumors. The m6A modification of RNA mainly consists of three parts: methyltransferase complex (writer), demethyltransferase (eraser) and recognition protein (reader). Writer mainly consists of methyltransferase 3 (METTL3), methyltransferase 14 (METTL14) and Wilms tumor 1-associating protein (WTAP).^100^ Recently, METTL16, KIAA1429, RNA-binding motif protein 15 (RBM15) and Zinc finger CCC domain protein 13 (ZC3H13) also have been found to have methyltransferase activity.^101,102^ The eraser mainly consists of FTO and ALKBH5. These two genes reversibly regulate the m6A level of RNA. Reader proteins mainly include YTHDF1/2/3, YTHDC1/2, and some RNA-binding proteins, including insulin-like growth factor 2 binding protein 1/2/3 (IGF2BP1/2/3), RNA-bound ELAV protein 1 (ELAVL1, also known as HuR), heterogeneous ribonucleoproteins (HNRNPs), FMR1 (Fragile X mental retardation 1) (Fig. 3).

As the most extensive post-transcriptional modification of RNA, m6A affects the splicing, stability, export and translation of RNA.^103^ Thus, m6A modification of RNA has a huge impact on the protein translation process of tumor cells.^15,102,104^ As the main methyltransferase, METTL3 stimulates the initiation of translation by increasing the recovery of eIF3, resulting in translation of target genes including epidermal growth factor receptor (EGFR) and Hippo pathway effector TAZ.^105^ METTL14, a key component of the m6A methyltransferase complex, is highly expressed in acute myeloid leukemia (AML). METTL14 increases m6A modification of MYB/MYC, promotes the protein translation, and maintains AML stem/initial cell self-renewal.^106^ In response to stress, such as amino acid starvation, the retranslation initiation of ATF4 is not mediated by the eIF2α signal but relies on m6A modification in the 5′UTR, which can control ribosome scanning and subsequent selection of start codons. Consistently, demethyltransferase FTO promotes ATF4 protein translation.^104^ A long noncoding RNA antisense to FOXM1 (FOXM1-AS) promotes the interaction of ALKBH5 with FOXM1 mRNA, increased FOXM1 expression by demethylation leading to the proliferation of stem-like cells in glioblastoma.^107^ YTHDF1 recognizes m6A modified mRNA. High-throughput sequencing showed that the ribosomes loaded by YTHDF1 targeted RNA are directly proportional to the binding of YTHDF1. Furthermore, YTHDF1 interacts with translation initiation factors to directly accelerate translation of ribosomal-bound mRNA.^103,108,109^ For instance, Tao and his colleagues found the translation of EIF3C, a subunit of translation initiation factor EIF3, was increased by YTHDF1 in an m^6^A-dependent manner. Consequently, the overall translation output of ovarian cancer is triggered, accelerating the tumorigenesis and metastasis.^110^ In HeLa cells, sequencing results combined with experiments have shown that YTHDF3 can coordinate with YTHDF1 to regulate translation. The mechanisms include YTHDF3’s regulation on translation initiation without directly binding translation initiation factors, and cooperation with YTHDF1 to jointly regulate translation.^111,112^

In addition, leucine-rich pentapeptide repeat (LRPPRC) may also act as m6A recognition protein,^102^ affects mitochondrial protein translation.^113^ In fragile X syndrome, X fragile mental retardation protein (FMRP) inhibits translation by binding to the L5 protein on the 80S ribosome directly.^114^ In autism spectrum disorders, FMR1 affects RNA metabolism by recognizing m6A modifications, including protein translation.^115^ M6A modification can also affect tRNA conditions and translation extension steps, thus altering translation kinetics.^116^ However, the exact mechanism of these genes has not been studied in tumors. Most of the m6A modifications of mRNA and small noncoding RNAs depend on the sequences and structures of mRNAs to work, and they will inevitably have a competitive or cooperative relationship in the translation process of mRNAs.

Collectively, the abnormal regulation of m6A methylation is closely related with the development of cancers ranging from metabolism, cell self-renewal, differentiation, and metastasis. Increasing evidences show that m6A modification of mRNA plays a dual role in cancer. On the one hand, methyltransferase complex increases the m6A modification of oncogenes to promote cancer development. On the other hand, FTO and ALKBH5 also promote tumor progression by reducing the modification of target genes.^117,118^ Therefore, the exact role of m^6^A in tumors needs to be judged according to the cellular contexts.

### The effects of codons and tRNAs on protein translation in tumors

The factors discussed above, including RBPs, noncoding RNAs and RNA m6A modification, perform function by regulating the mRNA structure and translation initiation complex. In addition to those factors, codons are one of the factors that affect the translation extension. Different codons were translated at different speeds.^119^ For example, GAA was found translated with a rate of 21.6 codons while GAG at 6.4 codons per second.^120^ Codon extension mainly depends on the entry of tRNA into the ribosome A-site through anticodon recognition.^121^ Therefore, the abundance of tRNA also plays a role in translation efficiency. Growing evidence demonstrated that dysregulation of tRNA was involved in tumor progression. Compared with normal cells, breast cancer cells have a 3-fold increase in nuclear-encoded tRNA expression and a 5-fold increase in mitochondrial-encoded tRNA to facilitate the translation of a subset of regulatory genes.^122^ For example, tRNAGluUUC and tRNAArgCCG were upregulated in breast cancer and promoted metastasis by increasing the ribosome occupancy of transcripts enriched for their cognate codons.^123^ Further researches identified two different subsets of tRNA pool between proliferation and differentiation, of which one is induced in proliferating cells and inhibited in other cases, and the other exhibiting the opposite characteristics. Subsequently, if this program hijacked by tumor cells to selectively upregulate proliferative tRNA, it will likely promote the translation of precancerous transcripts.^124^ In the future, tRNA may serve as a biomarker for cancer.

## Conclusion

Translation is an essential procedure for the protein expression, in this review we summarized the common factors affecting this procedure, including translation initiation factors, RBPs, noncoding RNAs (microRNAs, lncRNAs and circRNAs), and RNA m6A modification (Fig. 1). In addition, codons and tRNAs play important role on the translation elongation rate. The past decades have witnessed the rapid development of experimental techniques for research of translation, such as isotope or puromycin labeling,^125–128^ polysome profiling experiments using ultracentrifugation and density gradient separation, etc.,^129–131^ and these up-to-date techniques paved a wider path for researchers to investigate the potential molecular mechanism of tumor biology. After detecting abnormal translation in tumorigenesis induced by the above influencing elements, m6A and noncoding RNAs may potentially contribute to the prediction of the prognosis of tumor patients and the clinical application of antitumor therapy. Because of the widespread existence of RBPs, noncoding RNAs, m6A modification and tRNAs in eukaryotic cell, clarifying their relationship with aberrant protein translation is of great significance for the tumor treatment.
